# The Traditional Chinese Medicine Hua Tuo Zai Zao Wan Alleviates Atherosclerosis by Deactivation of Inflammatory Macrophages

**DOI:** 10.1155/2022/2200662

**Published:** 2022-03-28

**Authors:** Zhihua Yu, Xuanlu Zheng, Chenghui Wang, Chuan Chen, Na Ning, Danting Peng, Te Liu, Weidong Pan

**Affiliations:** ^1^Shanghai Geriatric Institute of Chinese Medicine, Shanghai University of Traditional Chinese Medicine, Shanghai 200031, China; ^2^Department of Neurology, Shuguang Hospital Affiliated to Shanghai University of Traditional Chinese Medicine, Shanghai 200031, China; ^3^Guangzhou Baiyunshan Qixing Pharmaceutical Co.,Ltd, Guangzhou 510530, China

## Abstract

**Introduction:**

Positive effects have been observed when the traditional Chinese medicine Hua Tuo Zai Zao Wan (HTZZW) has been used for the treatment of atherosclerosis (AS), although with an unclear mechanism.

**Methods:**

ApoE-/- C57/BALB mice were used to determine the efficacy of HTZZW by blood lipid biochemical analysis and histopathology H&E staining. qPCR and western blot were used to determine the expression of METTL3/14 and NF-*κ*B.

**Results:**

High-fat diet-fed ApoE-/- mice that consumed HTZZW exhibited significantly smaller plaque areas and significantly decreased unstable collagen areas in the aortic arch as well as significantly lower blood levels of total cholesterol, triglycerides, high-density lipoprotein cholesterol, and low-density lipoprotein cholesterol compared with the control group. Consumption of HTZZW significantly decreased the proportion of M*φ*1 in the peripheral blood. HTZZW not only inhibited the expression of m6A methyltransferases METTL14, METTL3, and overall RNA methylation level, but it also decreased the m6A modification level on specific sites of NF-*κ*B mRNA.

**Conclusion:**

HTZZW significantly alleviated the progression of AS by regulating the expression of the m6A methyltransferases METTL14 and METTL3 in macrophages, eliminating m6A modifications of NF-*κ*B mRNA, influencing the stability of NF-*κ*B mRNA, and ultimately resulting in the deactivation of inflammatory macrophages.

## 1. Introduction

Atherosclerosis (AS) is a chronic disease characterized by lipid accumulation, apoptosis and necrosis, smooth muscle cell proliferation, and local inflammation [1–4]. It may serve as a key pathological basis for cardiovascular and cerebrovascular diseases such as coronary heart disease and cerebral stroke [5–8]. The pathogenesis of AS is extremely complex, and previous studies have indicated that inflammation and lipid infiltration are closely related to vascular endothelial cell senescence and dysfunction [2, 3, 6, 9]. Reverse cholesterol transport (RCT) plays a pivotal role in plaque and necrotic core development in AS, while cytokines and chemokines can cause damage to endothelial cells and smooth muscle cells, thereby increasing the number of macrophages (M*φ*), which has an atherosclerosis-promoting effect. In addition, macrophages also play a role in the regulation of inflammation, which further promotes the development of AS [10, 11].

Many studies have indicated that Chinese herbal medicine significantly affects AS by delaying its onset and progression [1, 7]. The traditional Chinese medicine (TCM) compound formulation Hua Tuo Zai Zao Wan (HTZZW), which effectively promotes blood circulation and eliminates blood stasis, is used in China for the treatment of stroke sequelae such as hemiplegia, facial paralysis, and dysarthria. Its main constituents include Chuanxiong (the dry rootstock of *Ligusticum chuanxiong* Hort), Wuzhuyu (the nearly ripe, dried fruit of *Evodia rutaecarpa* (Juss.) Benth), and borneol. Although the use of HTZZW for the abovementioned conditions is well documented, its effect on AS and mechanism of action has not been reported in the current literature.

When methylation of the adenosine base at the nitrogen-6 (N-6) position in RNA occurs, N-6 methyladenosine (m6A) is formed. This modification, which is common in the mRNA of most eukaryotes (ranging from yeast, plants, and fruit flies to mammals) and viruses, plays a key regulatory role in posttranscriptional mRNA regulation and metabolism [12–16]. The m6A methyltransferases METTL14 and METTL3 form the stable m6A methyltransferase complex in a 1 : 1 ratio and perform RNA m6A modifications as a “writer” [5, 17–19], while the fat mass and obesity-associated (FTO) protein acts as an “eraser” to eliminate RNA m6A modifications [15, 17–19]. Therefore, RNA m6A modifications are dynamic and reversible enzymatic reactions [15, 17–19]. Some studies have suggested that RNA m6A modifications can enhance the stability, transcriptional activity, and translational activity of mRNA; promote tumorigenesis and tumor invasion; and increase stem cell reprogramming efficiency [16–18, 20, 21]. However, the changes and mechanism of action of RNA m6A modifications during the development of AS have not yet been elucidated.

Based on the evidence previously described, in the present study, we developed a mouse model of acquired AS by feeding ApoE^−/−^ mice with a high-fat diet. We then treated the mice with HTZZW to test the hypothesis that HTZZW alleviates AS progression by regulating m6A modification levels on NF-*κ*B mRNA, thereby influencing NF-*κ*B mRNA stability and decreasing M*φ* activity and inflammatory cytokine release.

## 2. Materials and Methods

All studies were performed in accordance with the Declaration of Helsinki, the Guidelines for Animal Studies of the University of Shanghai University of Traditional Chinese Medicine, and the National Institutes of Health of China. The committee of animal handling of the University of Shanghai University of Traditional Chinese Medicine also approved the experimental procedures used.

### 2.1. Animal Grouping and Drug Intervention

Forty male apolipoprotein E knockout (ApoE^−/−^) C57/BALB mice (SPF grade, 6–8 weeks old, body mass of 30 ± 5 g) were purchased from the Shanghai Research Center for Model Organisms (License No. SCXK (Shanghai) 2017-0004). HTZZW (License No. 17195) was purchased from Guangzhou Bai Yun Shan Qi Xing Pharmaceutical Co., Ltd. (Guangzhou, China). The ApoE^−/−^ C57/BALB mice were randomly assigned to four groups with 10 mice each after 1 week of acclimatization with normal feed: the blank control group (normal diet), the saline group (high-fat diet + equivalent volume of saline), the moderate-dose HTZZW group (HTZZW (M); high-fat diet + 8 g/kg HTZZW), and the low-dose HTZZW group (HTZZW (L); high-fat diet + 4 g/kg HTZZW). Drug intervention was performed for 12 weeks. The study was approved by the Ethics Committee at the Shanghai Institute of Geriatrics (SHAGESYDW2017008). All experiments were performed in accordance with China National Science and Technology Commission animal laboratory regulations.

### 2.2. Hematoxylin and Eosin (H & E) Staining

H & E staining was performed to observe the pathological morphology of aortic tissue obtained from the mice. After fixing the aortic tissue of each mouse in 10% formaldehyde, the aortic arch located 0.5 cm away from the aortic root was removed, dehydrated using the standard procedure, embedded in paraffin, and continuously sectioned starting from the aortic root (5-*μ*m thickness). The sections were stained using H & E to observe the pathological morphology under an optical microscope.

### 2.3. Masson Staining

Paraffin-embedded sections containing atherosclerotic plaques at the aortic root were sectioned, dewaxed, washed with double-distilled water for 5 min, and stained with hematoxylin for 5–10 min for nuclei staining. The stained sections were then thoroughly washed with water, stained with Masson's ponceau-fuchsin solution for 6–10 min, soaked in 2% aqueous glacial acetic acid for 5 s, differentiated in 1% aqueous phosphomolybdic acid for 3–5 min, directly stained with aniline blue for 5 min, soaked in 0.2% aqueous glacial acetic acid for several seconds, cleared with xylene, sealed, and photographed.

### 2.4. Fluorescence-Based Reverse Transcription Quantitative PCR (qRT-PCR)

Total RNA was extracted from the cells of various control and treatment groups using TRIzol reagent (Invitrogen) in accordance with the manufacturer's instructions. After treatment with DNase I (Sigma-Aldrich), the total RNA was quantified and reverse transcribed into cDNA using the ReverTra Ace-*α* First Strand cDNA Synthesis Kit (Toyobo). qRT-PCR was performed using a RealPlex4 real-time PCR detection system (Eppendorf) with SyBR Green RealTime PCR Master Mix (Toyobo) as the fluorescent dye for nucleic acid amplification. The qRT-PCR conditions were as follows: 40 amplification cycles of denaturation at 95°C for 15 s, annealing at 58°C for 30 s, and extension at 72°C for 42 s. The relative gene expression levels were determined using the 2^−ΔΔCt^ method, with ΔCt = Ct_genes−Ct_18sRNA and ΔΔCt = ΔCt_all_groups−ΔCt_blankcontrol_group. The mRNA expression levels were normalized using 18s rRNA. The primers used during amplification were as follows: mMettl3-F: 5′-GACTCTGGGCACTTGGAT-3ʹ; mMettl3-R: 5′-GTTGTGCTGGGCTTAGGG-3ʹ; mMettl14-F: 5′-GAACCGTGAAGCGAAGCA-3ʹ; mMettl14-R: 5′-AGCCTGGCCTGATAGTGC-3ʹ; mFto-F: 5′-AGGATGAAAGTGAGGACGAG-3ʹ; mFto-R: 5′-TGGTGAAGAGGGATTGTTA-3ʹ; m18SrRNA-F: 5′-AGGGGAGAGCGGGTAAGAGA-3ʹ; m18SrRNA-R: 5′-GGACAGGACTAGGCGGAACA-3ʹ.

### 2.5. Western Blotting

Total protein isolated from the various groups was subjected to denaturing electrophoresis using a sodium dodecyl sulfate-polyacrylamide gel electrophoresis (SDS-PAGE) system with a 12% gel and subsequently transferred to a polyvinylidene fluoride (PVDF) membrane (Millipore). After blocking and washing, the membrane was incubated with the primary antibody at 37°C for 45 min. The membrane was then thoroughly washed and incubated with the secondary antibody at 37°C for 45 min. After washing four times with Tris-buffered saline containing Tween-20 (TBST) for 14 min each time at room temperature, the membrane was treated with an enhanced chemiluminescence (ECL) reagent (Pierce Biotechnology) and exposed (Sigma-Aldrich).

### 2.6. Immunofluorescence Staining

Five mice were randomly selected from each group. From each mouse, a specimen containing plaques at the aortic root was resected, dehydrated, cleared, embedded in paraffin, sectioned, dewaxed, and rehydrated. After treatment with citrate buffer in a boiling water bath for 10 min for antigen retrieval, the specimen was cooled to room temperature, subjected to the same treatment again, and again cooled to room temperature. Blocking was performed at room temperature for 2 h using a 5% bovine serum albumin (BSA) blocking solution. The corresponding goat anti-rabbit polyclonal antibody was diluted to 1 : 500, and blocking was performed for 1 h. After overnight shaking at 4°C, the specimen was washed with phosphate-buffered saline (PBS) for 15 min. Fluorescein isothiocyanate (FITC)-conjugated anti-mouse IgG monoclonal secondary antibody was diluted in a blocking solution at a 1 : 500 ratio. Sections were incubated with a secondary antibody at room temperature for 1 h. After washing with PBS for 45 min, the specimen was mounted using an antifade medium containing 4ingperature for 1 h. The dary antibody was diluted in a blocking solution at the aortic root resected, dehydrated, cleared, and embedded in pa.

### 2.7. Blood Lipid Testing

Peripheral blood was collected from the mice. Blood samples were incubated at 4°C for 4 h and centrifuged at 10,000 rpm for 10 min at 4°C. The supernatant of each centrifuged sample was collected, and total cholesterol (TC), triglycerides (TG), high-density lipoprotein cholesterol (HDL-C), and low-density lipoprotein cholesterol (LDL-C) in serum were tested in accordance with the instructions provided with the test kit.

### 2.8. Dot-Blotting

Different doses of genomic DNA from each group were spotted on the Hybond-N+ membrane, and then the spotted DNA was cross-linked to the membrane by the UV crosslinker. The membrane was blocked in 5% BSA and subsequently incubated with anti-5hmC antibody (CST) and horseradish peroxidase (HRP)-conjugated anti-mouse secondary antibody (CST), and finally developed with ECL reagents and exposed to imaging film.

### 2.9. RNA Immunoprecipitation (RIP)-PCR

RIP experiments were performed using the Magna RIP RNA-Binding Protein Immunoprecipitation Kit (Millipore, Bedford, MA). All the steps of RIP were performed as previously described [22, 23]. In brief, cells from all groups were lysed (500 *μ*L per plate) in a modified cell lysis buffer used for western blotting and IP (20 mM Tris, pH 7.5, 150 mM NaCl, 1% Triton X-100, 1 mM EDTA, sodium pyrophosphate, *β*-glycerophosphate, Na_3_VO_4_, and leupeptin) (Beyotime Institute of Biotechnology). After lysis, each sample was centrifuged to clear the insoluble debris and was then preincubated with 20 *μ*g of protein A agarose beads (Beyotime Institute of Biotechnology) by rocking for 30 min at 4°C, followed by centrifugation and transfer to a fresh 1.5 mL tube. The mouse anti-human Ago2 monoclonal antibody (1 : 100; Santa Cruz Biotechnology, CA, USA) was added, and the solution was incubated for 90 min before the readdition of 20 *μ*g of protein A agarose beads to capture the immune complexes. The agarose beads were then washed three times with ice-cold homogenization buffer. The specific primers were designed as follows: NF-*κ*B-m6A-F: 5′-GCTCCTAAGGTGCTGACA-3ʹ; NF-*κ*B-m6A-R: 5′-TCCGAAAGCGAGATAAAG-3ʹ.

### 2.10. Statistical Analysis

Each experiment was performed at least three times, and the data are shown as the mean-standard error where applicable. Differences were evaluated with a Student's *t*-test. A *P* value of less than 0.05 was considered statistically significant.

## 3. Results

### 3.1. HTZZW Effectively Alleviates Pathological Manifestations of as in ApoE^−/−^ Mice

**Figure 1:**
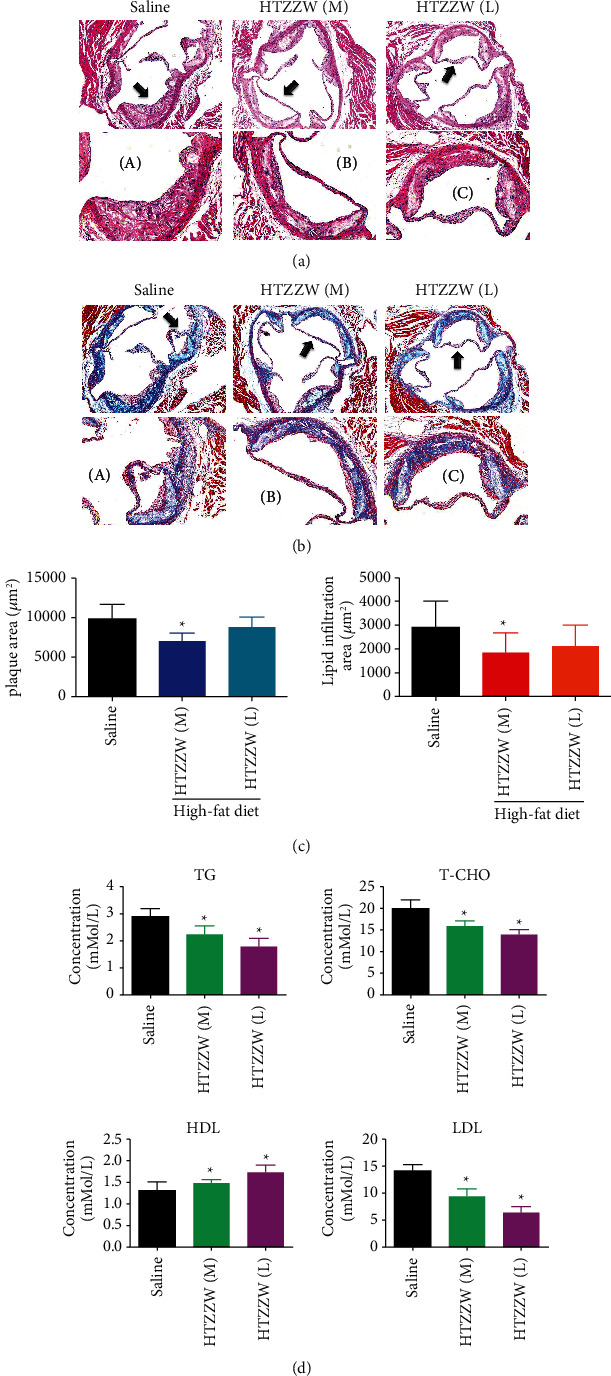
HTZZW effectively alleviated the pathological manifestations of AS in ApoE^−/−^mice. (a) H & E staining of the aortic roots of mice, magnification 100x. (a–c) High-power magnification of the regions indicated by arrows, magnification = 200x. (b) Masson staining of the aortic arch of mice, magnification 100x. (a–c) High-power magnification of the regions indicated by arrows, magnification 200x. (c) Statistical results of the plaque area and lipid infiltration area of the aortic arch in mice. ^*∗*^*P* < 0.05 vs. saline group; *t*-test; *n* = 8. (d) ELISA results of blood lipid testing. ^*∗*^*P* < 0.05 vs. saline group; *t*-test; *n* = 8.

H & E staining showed that the aortic roots of the saline group exhibited obvious lipid streak formation and foam cell aggregation, as well as large areas of lipid plaques, with a certain number of plaques being vulnerable, indicating the existence of the atheroma formation stage (Figure 1). However, compared with the saline group, there was a significantly smaller area of AS plaques and significantly reduced lipid deposition for the HTZZW (M) group (Figure 1). The results of Masson staining indicated that the proportion of collagen fibers in plaques at the aortic root of the saline group was significantly increased, while the proportion of unstable collagen fibers in plaques at the aortic root of the HTZZW (M) group was significantly decreased (Figure 1). In addition, the results of blood lipid tests performed on peripheral blood samples indicated that the serum TG, TC, LDL-C, and HDL-C levels of the HTZZW (M) group were significantly decreased compared to those in the blank control group (Figure 1). This indicated that a moderate dose of HTZZW significantly decreased vascular lipid deposition and peripheral blood lipid levels in mice with AS.

### 3.2. HTZZW Inhibits M*φ*1 Activity and Inflammatory Cytokine Release

**Figure 2:**
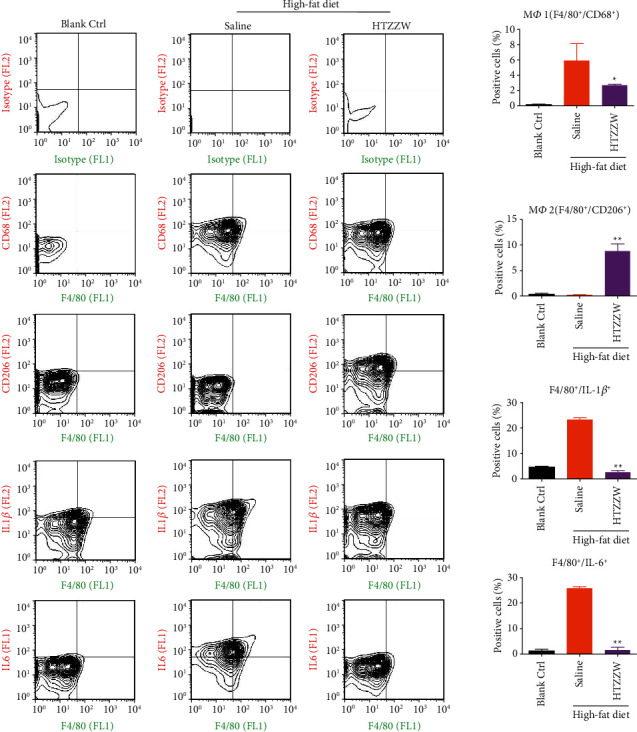
Flow cytometry results. HTZZW inhibited M*φ*1 activity and inflammatory cytokine release. ^*∗∗*^*P* < 0.01 vs. saline group; ^*∗*^*P* < 0.05 vs. saline group; *t*-test; *n* = 8.

Flow cytometry analyses indicated that the proportions of F4/80+/CD68+ (M*φ*1) and F4/80+/CD206+ (M*φ*2) cells in the peripheral blood of mice from the blank control group were extremely low, while the proportions of the two aforementioned types of cells were significantly increased in the saline group (Figure 2). However, after treatment of the AS mouse model using a moderate dose of HTZZW, the proportion of M*φ*1 significantly decreased, while the proportion of M*φ*2 significantly increased (Figure 2), indicating that HTZZW can significantly stimulate the conversion of M*φ*1 to M*φ*2 in AS mice. In addition, the proportions of F4/80+/IL − 1*β*+ and F4/80+/IL − 6+ significantly decreased after the treatment of AS mice with a moderate dose of HTZZW (Figure 2). These results suggest that HTZZW can significantly decrease macrophage activity and inflammatory cytokine release in AS mice.

### 3.3. HTZZW Influences the Overall RNA m6A Level in M*φ*1 Cells of as Mice

**Figure 3:**
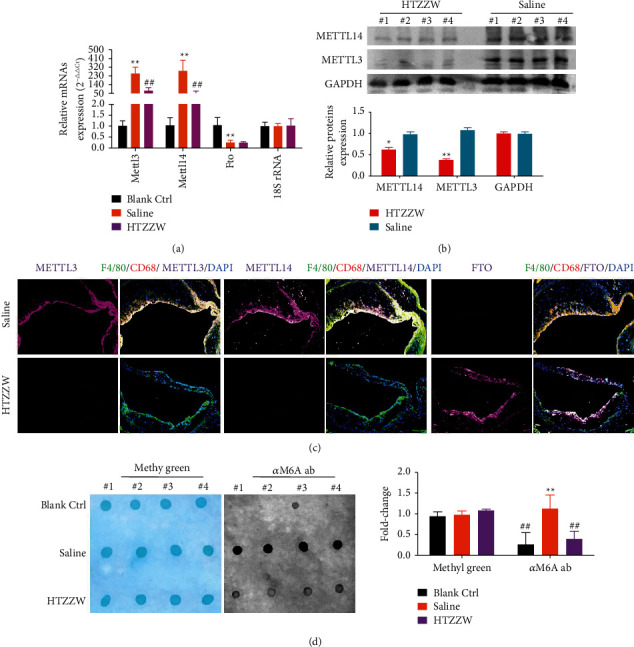
HTZZW influenced the expression of m6A methyltransferases and the overall RNA m6A level in the M*φ*1 of AS mice. (a) qPCR results of m6A methyltransferase expression levels in mRNA. ^*∗∗*^*P* < 0.01 vs. blank ctrl group; ^##^*P* < 0.01 vs. saline group; *t*-test; *n* = 8. (b) Western blotting results of RNA m6A methyltransferase expression levels. (c) Results of immunofluorescence staining of the aortic arch of mice. (d) Dot blot results of the overall RNA m6A level in mice. ^*∗∗*^*P* < 0.01 vs. blank ctrl group; ^##^*P* < 0.01 vs. saline group; *t*-test; *n* = 4.

Changes in expression levels of enzymes that control RNA m6A modifications in the various groups were measured. qPCR and western blotting indicated that Mettle3 and Mettle14 expression levels significantly increased, while the Fto expression level significantly decreased in the M*φ*1 cells of mice from the saline group (Figure 3). In contrast, the M*φ*1 of the HTZZW (M) group showed a significant decrease in the Mettle3 and Mettle14 expression levels and a significant increase in the Fto expression level (Figure 3), which was opposite to the results of the saline group. The results of immunofluorescence staining were also consistent with the results previously described (Figure 3). In addition, the results of dot blotting indicated that the overall RNA m6A level in the M*φ*1 of the HTZZW (M) group was significantly lower than that of the Saline group (Figure 3). These results demonstrated that HTZZW decreased the overall RNA m6A level in the M*φ*1 of AS mice by inhibiting the expression of RNA m6A methyltransferases.

### 3.4. HTZZW Inhibits NF-*κ*B RNA m6A Modifications and Results in Decreased NF-*κ*B RNA Expression

**Figure 4:**
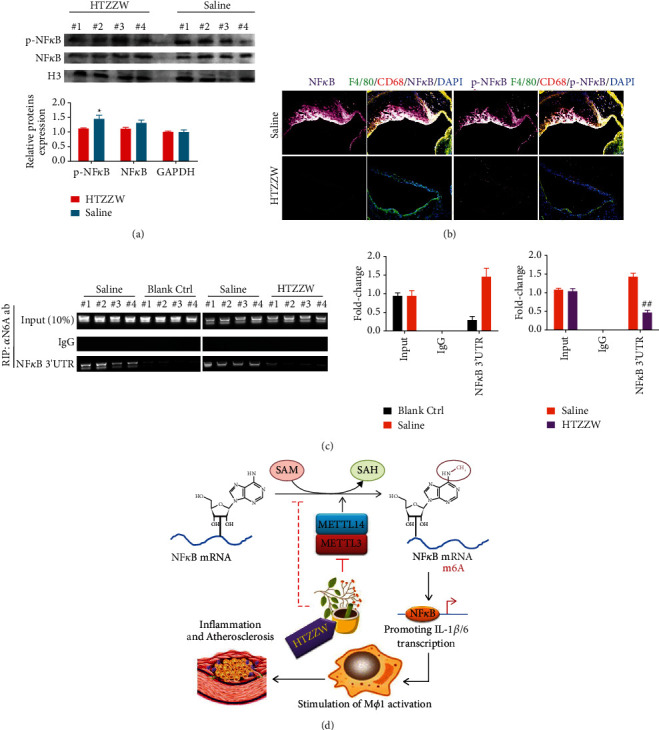
HTZZW inhibited NF-*κ*B RNA m6A modifications and induced a decrease in the expression level of NF-*κ*B RNA. (a) Western blotting results of the expression levels of NF-*κ*B and phosphorylated NF-*κ*B (p-NF-*κ*B) in cell nuclei. ^*∗∗*^*P* < 0.01 vs. saline group; *t*-test; *n* = 4. (b) Results of immunofluorescence staining of the aortic arch of mice. (c) RIP-PCR results. ^*∗∗*^*P* < 0.01 vs. blank ctrl group; ^##^*P* < 0.01 vs. saline group; *t*-test; *n* = 4. (d) The mechanism by which HTZZW alleviates inflammation and AS through the inhibition of NF-*κ*B RNA m6A modifications in M*φ*1.

Western blotting results indicated that the protein levels of total NF-*κ*B and phosphorylated NF-*κ*B (p-NF-*κ*B) in M*φ*1 nuclei of the saline group were significantly higher than those of the blank control group (Figure 4). However, the protein levels of total NF-*κ*B and p-NF-*κ*B in M*φ*1 nuclei of the HTZZW (M) group were significantly decreased (Figure 4). The results of immunofluorescence staining were consistent with the western blotting results (Figure 4). These results revealed that HTZZW inhibited the activation of NF-*κ*B in M*φ*1. Subsequently, the RIP-PCR results showed that complexes cross-linked to the anti-m6A antibody (*α* m6A ab) in the M*φ*1 of the saline group could be amplified to obtain specific products of the 3ʹ untranslated region (UTR) of NF-*κ*B mRNA (Figure 4). However, amplification of specific NF-*κ*B mRNA 3ʹ-UTR products was not observed for the complexes crossed-linked to *α* m6A ab in the M*φ*1 of the HTZZW (M) group (Figure 4). These results suggested that HTZZW inhibited m6A modifications at specific sites in the 3ʹ-UTR of NF-*κ*B mRNA, which resulted in decreased stability and expression of NF-*κ*B mRNA.

## 4. Discussion

Inflammation and abnormal lipid metabolism are important triggers of AS [2, 8, 24, 25], and macrophages are the key cells in AS plaques that are involved in lipid metabolism and inflammation [2, 8, 22, 23]. In a previous study, we showed that induction of the conversion of high cholesterol-related M*φ*1 to M*φ*2 significantly reduced inflammatory cytokine levels and foam cell formation in AS plaques [22]. This indicates that macrophages possessing an inflammatory phenotype aggravate the progression of AS. Further research revealed that the weakening of the polarity of M*φ*1 and alleviation of the disease progression of AS could be achieved by promoting the expression of transporters involved in reverse transcription (ATP-binding cassette transporter member 1 and ATP-binding cassette subfamily G member 1) and inhibiting the phosphorylation and activation of NF-*κ*B [22, 24, 25], which suggests that NF-*κ*B may be a potential target for the treatment of AS.

NF-*κ*B, which is pivotal in the regulation of cell responses, can be found in almost all animal cells and participates in cell responses to a variety of external stimuli, including cytokines, radiation, heavy metals, and viruses [22, 24–27]. It also plays a key role in the inflammatory and immune responses of cells [22, 24–27]. NF-*κ*B acts as a first responder to harmful cellular stimuli. There are many known activators of the NF-*κ*B pathway, including tumor necrosis factor-alpha (TNF-*α*), interleukin (IL)-1*β*, IL-2, IL-6, IL-8, and IL-12, induced nitric oxide synthase (iNOS), cyclooxygenase-2 (COX-2), chemokines, adhesion molecules, and colony-stimulating factors [22, 24–27].

Continued NF-*κ*B activation indirectly causes the polarization of M*φ*1 [22]. Many studies have investigated the activation and stability of NF-*κ*B at the transcriptional and translational levels using cells, including demethylation modifications in promoter regions and phosphorylation/acetylation modifications at certain sites of the NF-*κ*B protein [22, 24–27]. However, there are relatively few reports of methylation modifications in the NF-*κ*B mRNA.

RNAm6A refers to the methylation of the adenosine base at the nitrogen-6 (N-6) position in RNA. This modification, which is common in the mRNA of most eukaryotes (ranging from yeast, plants, and fruit flies to mammals) and viruses, plays a key role in posttranscriptional mRNA regulation and metabolism [12–16]. Meyer et al. and Dominissini et al. employed a method that combined m6A-specific methylated RNA immunoprecipitation with high-throughput sequencing to analyze human and mouse genes for the determination of transcriptome-wide RNA m6A distributions. RNA m6A was mainly distributed near the 3′-UTR of mRNAs and the stop codons of coding sequences (CDS). Furthermore, the distributions of m6A in human and mouse genes were found to be highly conserved. Other studies reported that RNA m6A enhanced mRNA stability [12, 13, 17, 20], and further research showed that m6A peaks were mainly enriched near the stop codons, 3′-UTRs, and long exons of mRNA, with the major conserved sequences being G (m6A) C (70%) and A (m6A) C (30%) [12, 14, 28].

Based on the results previously described, we measured the m6A modifications at specific regions in the 3′-UTR of NF-*κ*B mRNA and found that the m6A level at specific regions in the 3′-UTR of NF-*κ*B mRNA of macrophages of the wild-type (WT) group was extremely low, while the corresponding level was significantly increased in high-fat diet-fed ApoE^−^/^−^mice. We deduced that high methylation modification levels at the specific sites in NF-*κ*B mRNA resulted in its stable translation and expression. However, the TCM compound formulation HTZZW eliminated these m6A modifications, with the potential mechanism being the inhibition of the expression of the m6A methyltransferases METTL14 and METTL3 in macrophages. In addition, our experimental results also demonstrated that HTZZW significantly inhibited M*φ*1 polarization and significantly reduced the activity of inflammatory cytokine release, plaque area, and unstable collagen accumulation in the aortic arch as well as blood lipid levels in AS mice.

## 5. Conclusion

From the various aspects of phenotype, pathology, and epigenetics, the present study showed that the TCM compound formulation HTZZW significantly alleviated the progression of AS. Additionally, HTZZW exerted its effect via epigenetic regulation. It could regulate the expression of the m6A methyltransferases METTL14 and METTL3 in macrophages, thereby eliminating m6A modifications at specific regions in the 3′-UTR of NF-*κ*B mRNA, which influences the stability of NF-*κ*B mRNA and ultimately results in the deactivation of inflammatory macrophages.

## Data Availability

The data are available from the corresponding author on reasonable request.

## References

[1] Liu Q., Li J., Hartstone-Rose A. (2015). Chinese herbal compounds for the prevention and treatment of atherosclerosis: experimental evidence and mechanisms. *Evidence-Based Complementary and Alternative Medicine*.

[2] Bories G. F. P., Leitinger N. (2017). Macrophage metabolism in atherosclerosis. *FEBS Letters*.

[3] Wong B. W., Marsch E., Treps L., Baes M., Carmeliet P. (2017). Endothelial cell metabolism in health and disease: impact of hypoxia. *The EMBO Journal*.

[4] Zhang Y., Ren P., Kang Q. (2017). Effect of tetramethylpyrazine on atherosclerosis and SCAP/SREBP-1c signaling pathway in ApoE(-/-) mice fed with a high-fat diet. *Evidence-Based Complementary and Alternative Medicine*.

[5] Seimon T. A., Nadolski M. J., Liao X. (2010). Atherogenic lipids and lipoproteins trigger CD36-TLR2-dependent apoptosis in macrophages undergoing endoplasmic reticulum stress. *Cell Metabolism*.

[6] Xu H., Shi D., Chen K. (2012). Atherosclerosis: an integrative east-west medicine perspective. *Evidence-Based Complementary and Alternative Medicine*.

[7] Shen D. Z., Xin S. L., Chen C., Liu T. (2013). Effect of atorvastatin on expression of TLR4 and NF-kappaB p65 in atherosclerotic rabbits. *Asian Pacific Journal of Tropical Medicine*.

[8] Chen Z., Xu H. (2014). Anti-inflammatory and immunomodulatory mechanism of tanshinone IIA for atherosclerosis. *Evidence-Based Complementary and Alternative Medicine*.

[9] Wong W. Protected from atherosclerosis by TFEB. *Science*.

[10] Favari E., Chroni A., Tietge U. J., Zanotti I., Escola-Gil J. C., Bernini F. (2015). Cholesterol efflux and reverse cholesterol transport. *Handbook of Experimental Pharmacology*.

[11] Rohatgi A., Khera A., Berry J. D. (2014). HDL cholesterol efflux capacity and incident cardiovascular events. *Northern Engineer*.

[12] Meyer K. D., Saletore Y., Zumbo P., Elemento O., Mason C. E., Jaffrey S. R. (2012). Comprehensive analysis of mRNA methylation reveals enrichment in 3′ UTRs and near stop codons. *Cell*.

[13] Dominissini D., Moshitch-Moshkovitz S., Schwartz S. (2012). Topology of the human and mouse m6A RNA methylomes revealed by m6A-seq. *Nature*.

[14] Yue Y., Liu J., He C. (2015). RNA N6-methyladenosine methylation in post-transcriptional gene expression regulation. *Genes & Development*.

[15] Jia G., Fu Y., Zhao X. (2011). N6-methyladenosine in nuclear RNA is a major substrate of the obesity-associated FTO. *Nature Chemical Biology*.

[16] Chen T., Hao Y. J., Zhang Y. (2015). m(6)A RNA methylation is regulated by microRNAs and promotes reprogramming to pluripotency. *Cell Stem Cell*.

[17] Tang C., Klukovich R., Peng H. (2018). ALKBH5-dependent m6A demethylation controls splicing and stability of long 3’-UTR mRNAs in male germ cells. *Proc Natl Acad Sci U S A*.

[18] Chen M., Wei L., Law C. T. (2018). RNA N6-methyladenosine methyltransferase-like 3 promotes liver cancer progression through YTHDF2-dependent posttranscriptional silencing of SOCS2. *Hepatology*.

[19] Liu J., Yue Y., Han D. (2014). A METTL3-METTL14 complex mediates mammalian nuclear RNA N6-adenosine methylation. *Nature Chemical Biology*.

[20] Wang X., Lu Z., Gomez A. (2014). N6-methyladenosine-dependent regulation of messenger RNA stability. *Nature*.

[21] Barbieri I., Tzelepis K., Pandolfini L. (2017). Promoter-bound METTL3 maintains myeloid leukaemia by m(6)A-dependent translation control. *Nature*.

[22] Dou F., Chen J., Cao H. (2019). Anti-atherosclerotic effects of LXRalpha agonist through induced conversion of M1 macrophage to M2. *American Journal of Translational Research*.

[23] VanderBurgh J. A., Reinhart-King C. A. (2018). The role of age-related intimal remodeling and stiffening in atherosclerosis. *Advances in Pharmacology*.

[24] Ghosh S., Karin M. (2002). Missing pieces in the NF-kappaB puzzle. *Cell*.

[25] Napetschnig J., Wu H. (2013). Molecular basis of NF-kappaB signaling. *Annual Review of Biophysics*.

[26] Sun J., Huang P., Liang J. (2017). Cooperation of Rel family members in regulating Abeta1-40-mediated pro-inflammatory cytokine secretion by retinal pigment epithelial cells. *Cell Death & Disease*.

[27] Gong Z., Liu T., Wan Y. (2014). Decreased c-rel activation contributes to aberrant interleukin-2 expression in CD4(+)T cells of aged rats. *Molecular Immunology*.

[28] Bartosovic M., Molares H. C., Gregorova P., Hrossova D., Kudla G., Vanacova S. (2017). N6-methyladenosine demethylase FTO targets pre-mRNAs and regulates alternative splicing and 3′-end processing. *Nucleic Acids Research*.

